# Gastrointestinal Dysautonomia: A Rare Immune-Related Adverse Event That Requires Early Recognition and High-Dose Immunosuppression

**DOI:** 10.7759/cureus.92896

**Published:** 2025-09-22

**Authors:** Benjamin Norton, Stefan Sennfalt, Ross Nortley, Peter Hill, Paul Nathan, Valeria Iodice, Natalie Zarate-Lopez, Aisling Carr

**Affiliations:** 1 Gastroenterology, Digestive Disease and Surgery Institute, Cleveland Clinic London, London, GBR; 2 Gastroenterology, Centre for Obesity Research, University College London, London, GBR; 3 Neurology, National Hospital for Neurology and Neurosurgery, London, GBR; 4 Neuroscience, Neurosciences Institute, Cleveland Clinic London, London, GBR; 5 Nephrology, Acute Admissions Unit, Cleveland Clinic London, London, GBR; 6 Renal Medicine, Hammersmith Hospital, London, GBR; 7 Oncology, Mount Vernon Cancer Centre, Northwood, GBR; 8 Autonomic Unit, National Hospital for Neurology and Neurosurgery, London, GBR; 9 Gastroenterology, University College Hospital, London, GBR; 10 Neuromuscular Disease, National Hospital for Neurology and Neurosurgery, London, GBR; 11 Neuroscience, Institute of Neurology, University College London, London, GBR

**Keywords:** autoimmune autonomic neuropathy, dysautonomia, gastrointestinal dysautonomia, immune checkpoint inhibitors, immune-related adverse events (iraes), parenteral nutrition (pn)

## Abstract

Gastrointestinal (GI) immune-related adverse events (IrAEs) are relatively common following immune checkpoint inhibitor (ICI) therapy; however, GI dysautonomia is a very rare IrAE which manifests as pseudo-obstruction, gastroparesis, and/or achalasia. We present a case of seronegative GI dysautonomia secondary to pembrolizumab for metastatic melanoma, with symptoms of constipation, early satiety, and abdominal distension emerging within days of his second cycle, approximately five weeks after the initiation of therapy, which required temporary total parenteral nutrition. Concurrently, he developed autonomic features including blurred vision, dry mouth, and disabling orthostatic hypotension, suggesting a multi-system presentation. A full investigational work-up excluded a paraneoplastic cause, and autoantibodies (anti-Hu and anti-nicotinic acetylcholine receptor) were negative, indicating a seronegative presentation. Treatment with high-dose pulsed methylprednisolone, followed by a slow taper of prednisolone and initiation of maintenance mycophenolate, enabled recovery and re-establishment of oral nutrition. This case underscores that atypical GI IrAEs, such as dysautonomia, may necessitate early, high-intensity immunosuppression beyond current guideline-directed therapy. Rapid escalation of treatment may be life-saving and the only method to achieve functional and nutritional recovery.

## Introduction

Immune checkpoint inhibitors (ICI) are part of a range of cancer immunotherapeutic strategies that have revolutionised the modern treatment of many malignancies [1]. ICIs are monoclonal antibodies that target immunological receptors predominantly found on the surface of T-lymphocytes, including cytotoxic T-lymphocyte-associated protein 4 (CTLA-4), programmed death 1 (PD-1) and its ligand (PD-L1) [2]. These immune ‘checkpoints’ serve as negative regulatory pathways that tumours can exploit to suppress anti-tumour responses. Blocking these pathways restores and enhances T-lymphocyte activity, leading to a stronger anti-tumour response and improved overall survival in many cancers [3]. In only eight years, the number of US patients with cancer eligible for ICIs increased from 1.54% in 2011 to 43.6% in 2018 [4], which has since increased to 55.5% in 2023 [5]. With the surging use of ICI therapy, it is essential that clinicians, including gastroenterologists, are aware of the full spectrum of severe and potentially fatal adverse events that can occur with the use of these pharmacological agents. Side-effects due to ICIs are collectively known as immune-related adverse events (IrAEs) and are broadly classified into five grades of severity [6]. In patients with persistent or severe symptoms the principal treatment is with corticosteroids at a dose of 1-2 mg/kg/day of prednisolone (or equivalent dose) [6]. Gastrointestinal (GI) IrAEs are relatively common, particularly colitis that can present in over 20% depending on both the agent and use of combined therapy [7]. In contrast, GI dysautonomia is a very rare IrAE that can present with features of pseudo-obstruction, gastroparesis, and/or achalasia [8,9]. It is caused by inflammation of the enteric nervous system that leads to impaired autonomic regulation of GI motility, secretion, and sensory function [9]. Additionally, it can present in the context of a more generalised autonomic failure. Although rare, the presentation of GI dysautonomia is potentially life-threatening with higher risk of complications (e.g., perforation, aspiration), healthcare utilisation (e.g., enteral and parenteral nutrition), and ultimately, permanent disability or death. Consequently, we report our experience on the identification and management of a patient presenting with seronegative GI dysautonomia with multi-system involvement secondary to pembrolizumab for the treatment of metastatic melanoma. This is to highlight the utmost importance of maintaining a high degree of suspicion to enable early recognition and intensified immunosuppression, which led to the patient's functional and nutritional recovery.

## Case presentation

**Figure 1:**
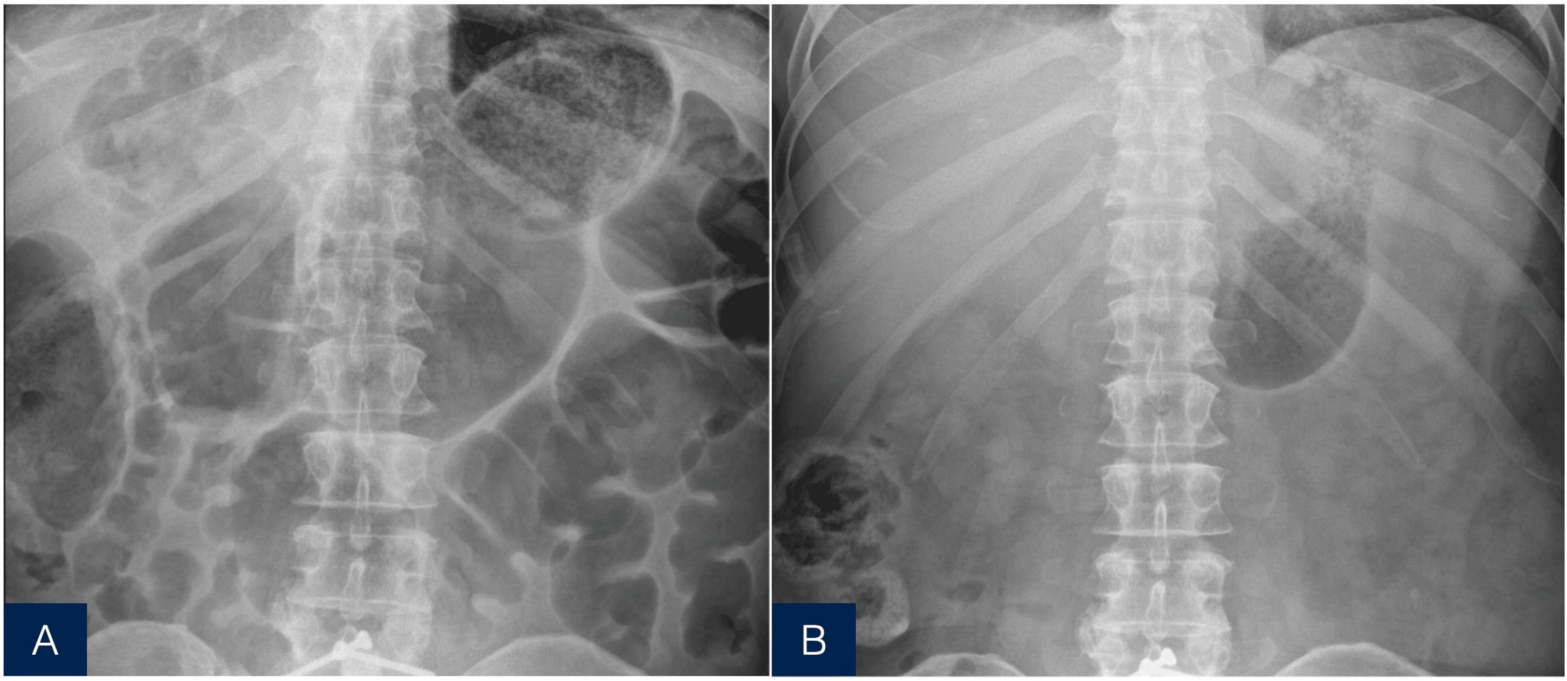
Plain film abdominal radiographs before and after treatment The plain film abdominal radiograph before treatment (A) showed markedly dilated intestines and gastric distention before escalation to methylprednisolone. After treatment (B), there was an improvement in dilatation and distension. This was two weeks after initiation of high-dose immunosuppression.

We present the case of a 58-year-old man with metastatic melanoma to the right inguinal lymph nodes following radical resection of a lower limb melanoma seven years ago. He was a non-smoker with no other past medical history, and his Eastern Cooperative Oncology Group (ECOG) performance status was zero. Following the detection of right external iliac lymphadenopathy, he commenced neoadjuvant pembrolizumab monotherapy. Approximately three weeks after his first dose, he developed mild constipation symptoms, managed with laxatives, and he proceeded to cycle two. Within days, he developed severe constipation, anorexia, early satiety, dysphagia, and abdominal distension. Initial investigations revealed a markedly dilated stomach and distended colon with no evidence of obstruction on abdominal x-ray and cross-sectional imaging (Figure 1A).

A barium swallow showed evidence of disorganised tertiary contractions in the distal third of the oesophagus. Concurrently, he had a painful, patchy sensory neuropathy and additional evidence of autonomic dysfunction including blurred vision, dry mouth, disabling orthostatic hypotension, and erectile dysfunction. Testing for anti-Hu and nicotinic acetylcholine receptor antibodies was negative, as was a full paraneoplastic screen for anti-neuronal autoantibodies. He was treated initially with seven days of oral prednisolone (60 mg daily), which was switched to intravenous hydrocortisone (100 mg every six hours) due to concerns of adrenal insufficiency with no clinical response. He then had intravenous immunoglobulin (IVIG) therapy at 0.4 g/kg/day for five days as a second line treatment, according to international IrAE management guidelines [6]. Despite two further five-day courses of IVIG at two and three months, his symptoms persisted. Due to his poor oral intake and significant weight loss (~8 kg) he required temporary total parenteral nutrition and inpatient care. A multi-specialty decision involving oncology, gastroenterology, and neurology lead to escalation of his therapy to methylprednisolone at 1 g/day for three days followed by 1 mg/kg oral prednisolone for four weeks. This was followed by a slow wean over months and then maintenance mycophenolate (1.5 g twice daily).

**Figure 2:**
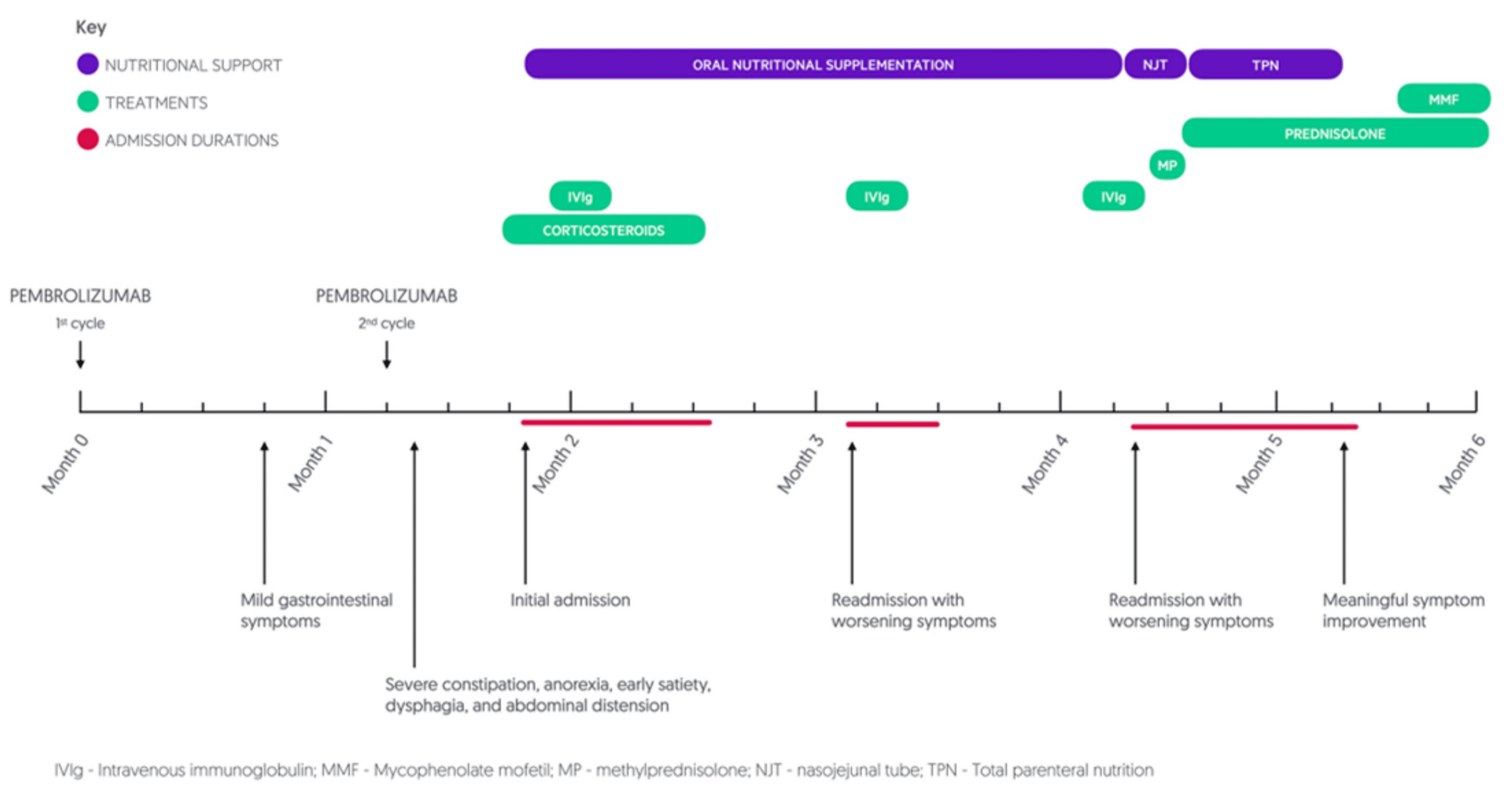
Timeline of clinical presentation The figure illustrates the chronology of symptoms, hospital admissions, nutritional interventions, and immunosuppressive treatments over the six-month course.

Four weeks after initiation of high-dose corticosteroids, he had achieved meaningful symptomatic improvement and could manage a normal diet and parenteral feeding was withdrawn. A full chronology of the presentation is shown in Figure 2.

Serial imaging demonstrated an improvement in the distended stomach and colon (Figure 1B). There was still a delay in gastric emptying observed on C^13^ breath testing (T½ = 155 minutes; normal <35-80 minutes) and body surface gastric mapping showed low frequency and high amplitude consistent with gastric dysrhythmia. There was also resolution in the neuropathic pain, allowing for discontinuation of amitriptyline and duloxetine, and improvement in orthostatic hypotension allowing for weaning from midodrine therapy. His melanoma remained in remission at his six-month follow up, and he is continuing with multi-disciplinary care review, including at the ICI-neurotoxicity service whilst symptoms are controlled on maintenance mycophenolate. 

## Discussion

This case highlights the potentially devastating development of seronegative GI-predominant dysautonomia secondary to ICI therapy with pembrolizumab. Adequate control required escalation to high-dose immunosuppression with both pulsed methylprednisolone, prolonged high-dose oral prednisolone, and steroid-sparing maintenance therapy with mycophenolate, which represent more aggressive immunosuppression than typically required in the management of most cases of immune-related colitis. Colitis is the most frequently observed IrAE, occurring in approximately 5.7-22.0% of patients [7]. There is currently well-established guidance on its recognition, diagnosis, and treatment, which typically involves lower doses of corticosteroids (e.g. methylprednisolone 1 mg/kg/day) for moderate to severe cases, which may be escalated to second-line monoclonal antibody therapy (e.g. anti-tumour necrosis factor alpha) in refractory cases [6].

In contrast, GI dysautonomia is a rare manifestation, which is in line with previous reports on neurological IrAEs (N-IrAEs) that are known to be uncommon compared to other organ-specific toxicities. A meta-analysis encompassing 9208 patients revealed a prevalence of 3.8% in those receiving anti-CTLA-4 inhibitors, 6.1% in patients on anti-PD-1 inhibitors, and 12.0% in individuals receiving combination therapy [10]. Most of these events were mild and non-specific, with headache being the most common manifestation, accounting for approximately half of the cases. Serious neurological adverse events are rare, occurring in fewer than 1% of patients [10,11]. Specifically, the occurrence of autoimmune autonomic neuropathy (AAN), which is the broader neurological term that encompasses the gut-specific presentation of GI dysautonomia, is rarely reported in association with ICIs and remains poorly characterised.

Across the literature, reports of GI dysautonomia have been limited to a few case reports. Appelbaum et al. [8] reported a fatal case of GI dysautonomia following a single dose of ipilimumab plus nivolumab for metastatic Merkel cell carcinoma. This led to irreversible GI dysmotility and sepsis from perforation. Autopsy revealed diffuse loss of enteric ganglion cells without inflammation but with C4d deposition, suggesting a potentially antibody-mediated mechanism. This is important as we know AAN can be associated with anti-cancer autoantibodies such as anti-Hu, which target neuronal structures in paraneoplastic syndromes, although our case remained seronegative [12]. Tezuka et al. [9] reported two new cases of GI dysautonomia secondary to the combination of nivolumab and ipilimumab for squamous cell lung cancer and durvalumab for small cell lung cancer, respectively. Both cases presented with intestinal dysmotility, additional autonomic dysfunction and other ICI-induced adverse events, including encephalitis and hepatitis, at four and 23 weeks after treatment initiation, respectively. Early initiation of treatment led to a good response in the first case, whereas the other, with a more delayed presentation, died secondary to IrAE complications despite pulsed methylprednisolone administered to both patients. The authors also completed a review of the literature, highlighting 11 additional cases with an average onset around 67.4 days after treatment initiation. Despite immunomodulatory treatment, full recovery was limited, five patients died, and the paper highlighted the diagnostic challenge, limited treatment efficacy, and the need to consider dysautonomia as a serious N-IrAE.

On the contrary, GI side-effects are commonly observed across all cancer treatments and do not necessarily indicate a serious underlying IrAE secondary to ICI therapy [13]. In one phase III trial assessing the efficacy and safety of nivolumab alone or nivolumab combined with ipilimumab for the treatment of melanoma, diarrhoea was reported in 19.2-44.1%, nausea in 13.1-25.9%, vomiting in 6.4-15.3% patients, but there were no reports of constipation or pseudo-obstruction [14]. Another phase III study comparing pembrolizumab versus ipilimumab for the treatment of advanced melanoma reported similar adverse events including diarrhoea (16.9-22.7%), nausea (10.1-11.2%), and vomiting (1.8-5.5%), and also abdominal pain (1.8-5.9%) and constipation (1.8-2.5%) [15]. However, none of these reported adverse events were grade 3-5 in severity. This showed that while GI side-effects were common, the development of GI dysautonomia with a substantial impact on the patient (e.g. ≥grade 3 IrAE) was very rare, but the true incidence may be under-reported.

The first reason is the distinction between an acute pan-GI presentation and isolated symptoms. When onset is abrupt and involves multiple regions of the GI tract, suspicion of immune-mediated enteric nervous system involvement is higher. In contrast, isolated symptoms such as constipation, nausea, or dysphagia, especially without other autonomic features, are less likely to prompt consideration of GI dysautonomia. The second reason is overlap with N-IrAEs. The clinical picture may be dominated by another IrAE, particularly a neurological syndrome that itself is rare at <1% [11]. In such cases, GI dysfunction may be overlooked or simply grouped within the broader neurological presentation [9]. Third, patients may undergo initial treatment for an alternative IrAE early in the disease process (e.g., pneumonitis, colitis) with more standardised doses of corticosteroids that could under treat or attenuate the underlying neuronitis. For example, Wu et al. [16] reported a case of GI dysautonomia and Guillain-Barre syndrome secondary to ipilimumab, in which the patient was initially treated for severe enterocolitis with corticosteroids and infliximab before re-presenting with autonomic dysfunction.

Taken collectively, in the absence of a high-degree of suspicion or concurrent autonomic/neurological dysfunction, the diagnosis of GI dysautonomia may be under-reported and lead to symptomatic management of more traditional causes (e.g., opioid-induced constipation). This is important, because although very rare, the presentation of GI dysautonomia will increase with the rising use of ICI therapy [4], and patients will undoubtedly encounter non-specialist clinicians and gastroenterologists for the management of vague GI symptoms weeks, months, or even years after the initiation of immunotherapy.

Due to its rarity, there is a paucity of evidence for the management of GI dysautonomia in the literature with several case reports demonstrating poor outcomes complicated by diagnostic delay, inadequately low doses of corticosteroids, and development of secondary complications when combined with additional treatments including IVIG and plasma exchange [8, 9]. We note that, in our case, low dose corticosteroids with IVIG was not enough to induce remission. We are of the opinion that early initiation of high-dose immunosuppression (e.g., pulsed methylprednisolone) is essential to switch off the inflammatory response within the enteric nervous system to improve the chance of recovery and prevent long-term sequalae, akin to the management of N-IrAE where good functional recovery can be achieved with similar treatment regimens [17]. Therefore, with the widespread and increasing use of ICI therapy, we emphasise the need for clinicians, especially gastroenterologists, involved in the management of GI IrAEs, to recognise this potentially fatal adverse event. Maintaining a high index of suspicion is crucial for early detection and prompt initiation of treatment that should be guided by the multidisciplinary team.

## Conclusions

GI dysautonomia is a rare but potentially life-threatening IrAE following ICI therapy. Unlike more common GI toxicities such as colitis, this entity may require more aggressive and sustained immunosuppression to achieve resolution.

Clinicians should be aware of this rare complication and consider it in patients presenting with upper and/or lower GI dysmotility features, especially in the context of other autonomic symptoms. Early recognition and timely escalation of immunosuppressive therapy may improve outcomes and reduce the risk of irreversible enteric nervous system injury. Multidisciplinary collaboration is key to optimising care in such complex presentations and provides an opportunity for systematic reporting of similar cases. This would help build the evidence base and ultimately inform future guidelines for the management of rare GI IrAEs. 
